# Inference to shape parameter of Nash model based on dynamic transport properties of channel networks

**DOI:** 10.1016/j.heliyon.2022.e11320

**Published:** 2022-10-31

**Authors:** Joo-Cheol Kim, Yeo-Jin Yoon

**Affiliations:** a International Water Resources Research Institute, Chungnam National University, 99 Daehak-ro, Yuseong-gu, Daejeon, 34134, Republic of Korea; b Department of Disaster Safety & Fire, Konyang University, 121 Daehak-ro, Nonsan-si, Chungcheongnam-do, 32992, Republic of Korea

**Keywords:** Nash model, Channel network, Horton's ratios, Fractal dimension, Random walk

## Abstract

This study investigates the relations between the shape of hydrologic responses and the dynamic transport properties of channel networks within the framework of random walks on fractal networks, focusing on the shape parameter of Nash model. To this end, we evaluate the static fractal structures and the dynamic transport properties of various channel networks and, then, validate Liu's conjecture (1992) for the shape of hydrologic responses. In the context of random walks on fractal networks, the fractal dimensions of channel networks can directly connect the static structure to the dynamic transport properties of channel networks through Horton's law of drainage composition. It is observed that the peak coordinates of hydrologic responses would have a more intimate relation to the connectivity of channel networks than the conductivity of those. The characteristic times of hydrologic responses also tend to be related to the connectivity of channel networks. Thereby, the shape of hydrologic responses would be expected directly affected by the fractal dimension of channel networks in terms of their static structure, while interpreted a combined result of the conductivity and the connectivity of channel networks in terms of their dynamic transport properties. So, the runoff hydrographs of a river basin could be considered shaped by the fractal dimensions of its channel networks following the linear hydrologic system theory.

## Nomenclature

RB: Bifurcation Ratio of Channel Network
RL: Length Ratio of Channel Network
RA: Area Ratio of Channel Network
$N_{\omega }$: The Number of The $\omega^{th}$ Order Stream, ω=1,2,⋯Ω
$L_{\omega }$: Average Length of The $\omega^{th}$ Order Stream, ω=1,2,⋯Ω
$A_{\omega }$: Average Drainage Area of The $\omega^{th}$ Order Stream, ω=1,2,⋯Ω
L‖: Diffusion Length
K(L‖): Variable Diffusion Constant along $L_{\|}$
$\sigma \left(L_{\|}\right)$: Conductivity between Two Points Distant of $L_{\|}$ on Fractal Structure
D: Fractal Dimension of Channel Network as A Whole
dN: Topological Fractal Dimension of Channel Network
dL: Path Fractal Dimension of Channel Segment
dw: Spatial Diffusion Dimension (Walk Dimension)
ds: Spectral Dimension (Fracton dimension)
d: Euclidean Dimension of Substrates
〈r2(t)〉: Mean Squared Displacement of Random Walk
p(r,t): Distribution of Displacement at Time *t* for Random Walk
p(0,t): Return Probability of Random Walk after Time *t*
h(t): Instantaneous Unit Hydrograph (IUH)
P(s): Path Probability for Specific Flow Path *s*
$f_{\omega }\left(t\right)$: Probability Density Function (PDF) of Waiting Time for The $\omega^{th}$ Order Stream, ω=1,2,⋯Ω
α: Exponent of Anomalous Diffusion
θ: Scaling Exponent of Variable Diffusion Constant
μ: Conductivity (or Resistance) exponent
n: Shape Parameter of Nash Model
k: Scale Parameter of Nash Model
hp: Peak of h(t)
tp: Time to Peak of h(t)
tL: Basin Lag Time
kˆ: Dimensionless Scale Parameter of Nash Model
hˆp: Dimensionless Peak of h(t) for Geomorphologic IUH (GIUH)
tˆp: Dimensionless Time to Peak of h(t) for GIUH
tˆL: Dimensionless Basin Lag Time
v: Mean Flow Velocity
β: Product of $h_{p}$ and $t_{p}$ of Nash Model
$\beta_{G}$: Product of $h_{p}$ and $t_{p}$ of GIUH

## 1 Introduction

In hydrology, Nash model is one of the classical conceptual rainfall-runoff models for an instantaneous unit hydrograph (IUH) based on the cascade of equal linear reservoirs (Nash, 1957). This model is in the form of a probability density function (PDF) for the two-parameter gamma distribution and, thereby, very simple in structure. Accordingly, it is amenable to the mathematical manipulation for rainfall-runoff processes and the analytical derivation of the characteristic parameters for hydrologic responses such as the peak or time to peak of IUH (Rosso, 1984). It is also well known that the shape parameter of Nash model, more specifically the number of equal linear reservoirs, is not restricted to an integer. This might be reminiscent of fractional calculus for linear differential equations (Borthwick, 2010) and clearly indicates that linear reservoirs are not a physical component of river basins. Therefore, this parameter couldn't be directly related to the scale of river basins, so its physical meaning could be ambiguous.

Meanwhile, runoff generated on hillslopes is generally routed through channel networks organized by interwoven individual channel segments. So, since Horton's seminal works (Horton, 1945), an approach to the drainage structure of river basins has been conducted mainly focusing on the form of channel networks (Shreve, 1966; Smart, 1972; Strahler, 1952). In this regard, Rodríguez-Iturbe and Valdés (1979) develop a theory of geomorphologic instantaneous unit hydrograph (GIUH) by combining hydrologic responses with the basin geomorphology on the basis of Horton's law of drainage composition (Horton, 1945; Schumm, 1956). Especially, following GIUH theory, Nash model is parameterized with Horton's ratios by Rosso (1984) and, subsequently, applied to the various research on the geomorphology-based hydrologic responses of river basins (Bhunya et al., 2008; Choi et al., 2011). Nevertheless, it is still hard to find a clear explanation for the shape parameter of Nash model, more broadly the shape of the hydrologic responses of river basins.

This study aims to interpret the geomorphology-based hydrologic implication on the shape parameter of Nash model by analyzing its relations to the fractal structures of channel networks. As mentioned before, this parameter could be estimated by Horton's ratios with the help of GIUH theory (Bhunya et al., 2008; Rosso, 1984). It is also well known that Horton's ratios have a close relationship with the fractal dimensions of channel networks which characterize the degree of space-filling due to the meandering of channel segments and branching of channel networks (La Barbera and Rosso, 1989; Rosso et al., 1991; Tarboton et al., 1988). Especially, Liu (1992) categorizes the fractal properties of channel networks into the static structures and the dynamic transport properties of fractal networks and, then, emphasizes their hydrologic implications by comparing those with random aggregation structures of percolation clusters. In fact, river basins couldn't be considered a homogeneous pathway of runoff due to the coexistence of hillslopes and channels. Furthermore, there exist distinct characteristics of water particles' movement between hillslopes and channels in that the difference in flow velocity between those might be about 100 times (D'Odorico and Rigon, 2003). So, when considering water flow through channel networks at the basin scale the restriction to water particles' movement should be taken into account induced by the fractal geometry of channel networks. In the context of random walks on fractal networks (Alexander and Orbach, 1982; Sokolov, 2012), the dynamic transport properties of channel networks can be viewed as an indicator of the transport process through random networks featured by fractal geometry. It is well known that the dynamic transport properties of fractal networks consist of the spatial diffusion dimension and the spectral dimension, in that the former characterizes the conductivity of fractal networks while the latter the connectivity of fractal networks (Alexander and Orbach, 1982). Furthermore, it is noted that there is a certain relation between the static structures and the dynamic transport properties of fractal networks, which can be an avenue to approach the relations between the form of channel networks and their corresponding transport properties (Cates, 1984; Havlin et al., 1984; Orbach, 1986). In this study, we try to investigate the relations between the hydrologic responses of river basins and the fractal structures of channel networks, focusing on the shape parameter of Nash model and the dynamic transport properties of channel networks.

## 2 Fractal dimensions of channel networks

### 2.1 Static fractal structure of channel networks

#### 2.1.1 Horton's ratios

Strahler's stream ordering scheme (Strahler, 1952) is well known for assigning the topology of channel networks. The followings point out its main idea.

• Channels that originate at sources are defined to be the first order streams.
• When two streams of order *ω* join a stream of order ω+1 is generated.
• When two streams of different order join the channel segment immediately downstream has the higher order of the two combining streams.

Once the topology of a channel network with the highest order being *Ω* is determined according to Strahler's stream ordering scheme, Horton's law of drainage composition (Horton, 1945; Schumm, 1956) can be defined by

(1) $$R_{B}=\frac{N_{\omega -1}}{N_{\omega }};\;N_{\omega }=N_{\Omega }R_{B}^{\Omega -\omega }$$

(2) $$R_{L}=\frac{L_{\omega }}{L_{\omega -1}};\;L_{\omega }=L_{\Omega }R_{L}^{-\left(\Omega -\omega \right)}$$

(3) $$R_{A}=\frac{A_{\omega }}{A_{\omega -1}};\;A_{\omega }=A_{\Omega }R_{A}^{-\left(\Omega -\omega \right)}$$

where $R_{B}$, $R_{L}$, and $R_{A}$ refer to bifurcation, length, and area ratio respectively with $N_{\omega }$, $L_{\omega }$, and $A_{\omega }$ being the number, average length, and average drainage area of the $\omega^{th}$ order stream respectively.

#### 2.1.2 Traditional fractal dimensions of channel networks

Horton's ratios in Eq. (1) to Eq. (3) are intimately related to fractal dimensions of channel networks. La Barbera and Rosso (1989) suggest a relation of the fractal dimension $d_{N}$, the so-called topological dimension, based on Horton's law of drainage composition

(4) $$d_{N}=\frac{\ln R_{B}}{\ln R_{L}}$$

Eq. (4) reflects a scaling behavior of channel networks, in that linear channel segments would organize a kind of network structure to fulfill a planar basin by branching process. Furthermore, Rosso et al. (1991) propose another fractal dimension for channel segment, $d_{L}$, the so-called path dimension, in terms of Horton's law of drainage composition

(5) $$d_{L}=2\frac{\ln R_{L}}{\ln R_{A}}$$

where $d_{L}$ quantifies sinuosity of individual channel segments. Therefore, the fractal dimension of channel networks as a whole, *D*, (Tarboton et al., 1988) can be expressed by

(6) $$D=d_{L}d_{N}=2\frac{\ln R_{B}}{\ln R_{A}}$$

By comparing channel networks with artificial random aggregates such as percolation clusters, Liu (1992) derives the same relations as Eq. (4) to Eq. (6) for the static fractal structure of channel networks.

### 2.2 Dynamic transport properties of channel networks

#### 2.2.1 Random walks on fractal networks

Within the framework of anomalous diffusion on the free Euclidean space, the mean squared displacement of a random walk at time *t*, $\langle r^{2}\left(t\right)\rangle$, is expressed by

(7) $$\langle r^{2}\left(t\right)\rangle \propto t^{\alpha }$$

For α=1, Eq. (7) reduces to normal diffusion whereas it refers to sub-diffusion if α<1. By defining a diffusion length to be $L_{\|}={\langle r^{2}\left(t\right)\rangle }^{1/2}$, allowing for scaling of diffusion constant $K\left(L_{\|}\right)\propto L_{\|}^{-\theta }$ can give rise to

(8) $$\alpha =\frac{2}{2+\theta }$$

Based on Eq. (7) and Eq. (8), the fractal dimension of the random walk trajectory embedded in the free Euclidean space, $d_{w}$, can be derived as Eq. (9) in terms of *θ* (Gefen et al., 1983; Orbach, 1986)

(9) $$d_{w}=2+\theta$$

It is noted that $d_{w}=2$ for normal diffusion with θ=0. The conductivity *σ* between two points of a fractal structure follows a scaling law (Ben-Avraham and Havlin, 2000; Hendrick and Renard, 2016)

(10) $$\sigma \left(L_{\|}\right)\propto L_{\|}^{-\mu }$$

where *μ* is the conductivity (or resistance) exponent. It is well known that Einstein's relation links $\sigma \left(L_{\|}\right)$ to $K\left(L_{\|}\right)$ and the density of the substrates *ρ* (Ben-Avraham and Havlin, 2000; Gefen et al., 1983; Hendrick and Renard, 2016)

(11) $$\sigma \left(L_{\|}\right)\propto \rho K\left(L_{\|}\right)$$

So, *μ* can be expressed as Eq. (12) by comparing Eq. (10) with Eq. (11) (Hendrick and Renard, 2016)

(12) $$\mu =d_{w}-D+d-2$$

where *d* is the Euclidean dimension of the substrates. If considering an additional random walk occurring only within the random walk trajectory of normal diffusion in the case of d=2 in similar to channel networks, we have μ=θ because *D*, the fractal dimension of the first random walk trajectory, is universally 2. Thereby, we can infer the conductivity of water particles through the channel networks with the fractal dimension of *D* in the context of anomalous diffusion

(13) $$d_{w}=D+\mu$$

Meanwhile, for a random walk on the *d*-dimensional space, the distribution of *r* at time *t* follows Gaussian in the form of Eq. (14) (Sokolov, 2012)

(14) $$p\left(r,t\right)=\frac{1}{{\left(4\pi Kt\right)}^{d/2}}e^{-\frac{dr^{2}}{4\pi K}}$$

Therefore, the return probability of the random walk after time *t* scales as

(15) $$p\left(0,t\right)\propto t^{-\frac{d}{2}}$$

Eq. (15) refers to the probability for a random walker to be at the origin at time *t* so that it closely relates to the connectivity of the random walk trajectory. When considering an additional random walk limited to the first random walk trajectory again, $L_{\|}$ of the second random walk at time *t* can be expressed by Eq. (16)

(16) $$L_{\|}\propto t^{\frac{1}{d_{w}}}$$

Furthermore, the overall structure of the first random walk trajectory scales as Eq. (17)

(17) $$L_{\|}^{D}\propto t^{\frac{D}{d_{w}}}$$

Since p(0,t) of the second random walk on the first random walk trajectory is proportional to $L_{\|}^{-D}$, Eq. (15) can be converted into Eq. (18)

(18) $$p\left(0,t\right)\propto t^{-\frac{D}{d_{w}}}$$

Thereby, we can also infer the connectivity of the channel networks with the fractal dimension of *D* in the context of $d_{w}$

(19) $$d_{s}=2\frac{D}{d_{w}}$$

where $d_{s}$ replaces *d* in Eq. (15) to account for the limited motion of the second random walk on the first random walk trajectory.

#### 2.2.2 Additional fractal dimensions of channel networks

In contrast to Eq. (4) and Eq. (5), Liu (1992) also introduces the spatial diffusion dimension $d_{w}$ (also called the walk dimension) and the spectral dimension $d_{s}$ (also called the fracton dimension) (Alexander and Orbach, 1982) which are presented in the previous section, in order to describe the dynamic transport properties of channel networks. Following the argument of Havlin et al. (1984) that $\mu \propto D/d_{N}=d_{L}$, Liu (1992) converts Eq. (13) and Eq. (19) into

(20) $$d_{w}=d_{L}\left(d_{N}+1\right)=2\frac{\ln\left(R_{B}R_{L}\right)}{\ln R_{A}}$$

(21) $$d_{s}=2\frac{d_{N}}{d_{N}+1}=2\frac{\ln R_{B}}{\ln\left(R_{B}R_{L}\right)}$$

It is noted that, through Horton's law of drainage composition, Eq. (20) and Eq. (21) can directly connect the dynamic transport property of channel networks to their static structure represented by Eq. (4) and Eq. (5). Furthermore, it can be also seen that though $d_{w}$ in Eq. (20) is closely related to $d_{L}$, $d_{s}$ in Eq. (21) is independent of $d_{L}$ and, thereby, it is considered to be an intrinsic parameter of the network connectivity (Cates, 1984; Liu, 1992).

## 3 Hydrologic responses s of channel networks

### 3.1 Nash model

Nash model for IUH h(t) can be written the PDF of the two-parameter gamma distribution (Nash, 1957)

(22) $$h\left(t\right)=\frac{1}{k\Gamma \left(n\right)}{\left(\frac{t}{k}\right)}^{n-1}e^{-\frac{t}{k}}$$

where *n* is a shape parameter indicating the number of equal linear reservoirs in series while *k* is a scale parameter corresponding to the storage coefficient of a linear reservoir. In addition, Γ(⋅) denotes the gamma function, so that *n* needs not to be an integer. By taking the first derivative of Eq. (22), analytical relations can be derived for the peak $h_{p}$ and time to peak $t_{p}$ of h(t)

(23) $$h_{p}=\frac{{\left(n-1\right)}^{n-1}}{\Gamma \left(n\right)}e^{-\left(n-1\right)}\frac{1}{k}$$

(24) $$t_{p}=\left(n-1\right)k$$

It is noted that the product *β* of Eq. (23) and Eq. (24) results in an independent relation on *k*

(25) $$\beta =\frac{{\left(n-1\right)}^{n}}{\Gamma \left(n\right)}e^{-\left(n-1\right)}$$

Accordingly, several previous studies (Bhunya et al., 2008; Chavan and Srinivas, 2015; Rosso, 1984) regard Eq. (25) to be a salient characteristic for the shape of hydrologic responses.

### 3.2 GIUH

GIUH can be formulated within the framework of Strahler's stream ordering scheme Rodríguez-Iturbe and Valdés, 1979; Gupta et al., 1980)

(26) $$h\left(t\right)=\sum_{s}P\left(s\right){\left[f_{\omega }\left(t\right)⊛\cdots ⊛f_{\Omega }\left(t\right)\right]}_{s}$$

where P(s) is the path probability for a specific flow path *s* while the multiple terms in the square bracket represent the PDF of the water particles' travel time to the outlet through *s* with ⊛ being the convolution operator. Rodríguez-Iturbe and Valdés (1979) assume the PDF of waiting time, $f_{\omega }\left(t\right)$, to be the exponential distribution for any stream of order *ω* (=1,2,⋯,Ω)

(27) $$f_{\omega }\left(t\right)=\lambda_{\omega }e^{-\lambda_{\omega }t}$$

where $\lambda_{\omega }$ is the inverse of the mean waiting time within the stream of order *ω*. It is well known that though Eq. (26) can be extended to an arbitrarily large order of stream with the help of Eq. (27), those full formulations could be extremely complicated (Bhunya et al., 2008; Rosso, 1984). So, based on Horton's law of drainage composition, Rodríguez-Iturbe and Valdés (1979) develop the relations of $h_{p}$ and $t_{p}$ for GIUH by the regression analysis

(28) $$h_{p}=1.31R_{L}^{0.43}\frac{v}{L_{\Omega }}$$

(29) $$t_{p}=0.44{\left(\frac{R_{B}}{R_{A}}\right)}^{0.55}R_{L}^{-0.38}\frac{L_{\Omega }}{v}$$

where *v* is the mean flow velocity. In similar to Eq. (25), the product $\beta_{G}$ of Eq. (28) and Eq. (29) can give rise to an independent relation on both of *v* and $L_{\Omega }$

(30) $$\beta_{G}=0.58{\left(\frac{R_{B}}{R_{A}}\right)}^{0.55}R_{L}^{0.05}$$

Thereby, Rosso (1984) assumes that $\beta =\beta_{G}$ to suggest the geomorphologic relations for two parameters of Nash model

(31) $$n=3.29{\left(\frac{R_{B}}{R_{A}}\right)}^{0.78}R_{L}^{0.07}$$

(32) $$k=0.70{\left(\frac{R_{A}}{R_{B}R_{L}}\right)}^{0.48}\frac{L_{\Omega }}{v}$$

Rosso (1984) uses Eq. (25) which doesn't lend itself to an explicit relation of *n*. So, Eq. (31) and Eq. (32) have resulted from the regression analysis with the coherent unit of *v* and $L_{\Omega }$ in similar to Eq. (28) and Eq. (29). It can be seen that the product of Eq. (31) and Eq. (32) produces the first order of statistical moment for water particles' travel time to the outlet, $t_{L}$, in the context of Nash model

(33) $$t_{L}=nk=2.30{\left(\frac{R_{B}}{R_{A}}\right)}^{0.30}R_{L}^{-0.41}\frac{L_{\Omega }}{v}$$

In hydrology, $t_{L}$ in Eq. (33), the so-called basin lag time, is considered to be one of the most important characteristic times of the basin response to rainfall along with $t_{p}$ in Eq. (24) and Eq. (29) (Singh, 1988).

## 4 Materials and methodology

### 4.1 Liu's conjecture

Based on the already published data set for twelve channel networks in the Appalachian Plateau (Morisawa, 1962), Liu (1992) claims that though Eq. (20) and Eq. (21) are extremely different from Eq. (31) and Eq. (30) respectively in the context of their mathematical derivation, the values of *n*, $\beta_{G}$, and $n\beta_{G}$, in turn, tend to approximate the values of $d_{w}$, $d_{s}/2$, and *D*. So, he hypothesizes certain relations between the shape of hydrologic responses and the dynamic transport properties of channel networks

(34) $$n\approx d_{w}$$

(35) $$\beta_{G}\approx \frac{d_{s}}{2}$$

(36) $$n\beta_{G}\approx D$$

Eq. (34) to Eq. (36) stress that the dynamic transport properties of channel networks may have important roles in shaping hydrologic responses at the basin scale. We, thus, try to test the validity of Liu's conjecture more in detail by using the same data set used by him. Moreover, we add two more data sets previously published in the literature (Marani et al., 1991; Rosso et al., 1991), which are comprised of the twenty-five channel networks exploited by various authors. Table 1 lists the collection of data sets used in this study, where * denotes the anomalous values of $d_{L}$, $d_{N}$, and *D*, less than one or greater than two. Theoretically, channel networks are fractals embedded in a planar basin, so those fractal dimensions should be somewhere between one and two. Liu (1992) points out that the anomalous values with * may be due to factors such as the lithology of bedrocks and geological structures.

**Table 1:** The Published Data Set for the Various Channel Networks.

Source	Basin	*R*_*B*_	*R*_*L*_	*R*_*A*_	*d*_*L*_	*d*_*N*_	*d*_*w*_	*d*_*s*_	*D*	${\overset{ˆ}{h}}_{p}$	${\overset{ˆ}{t}}_{p}$	*β*_*G*_	*n*	$\overset{ˆ}{k}$	${\overset{ˆ}{t}}_{L}$	*nβ*_*G*_
Morisawa (1962)	Tar Hollow	4.13	3.55	5.26	1.53	1.12	3.23	1.06	1.71	0.63	0.86	0.54	2.98	0.43	1.27	1.61
	Home Creek	2.90	2.17	3.36	1.28	1.37	3.04	1.16	1.76	0.51	1.09	0.56	3.10	0.52	1.60	1.72
	Mill Creek	4.67	2.66	4.75	1.26	1.58	3.23	1.22	1.98	0.55	1.08	0.60	3.48	0.44	1.53	2.10
	Green Lick	4.41	3.32	5.12	1.47	1.24	3.29	1.11	1.82	0.61	0.92	0.57	3.18	0.42	1.35	1.81
	Beech Creek	3.80	2.61	4.05	1.37	1.39	3.28	1.16	1.91	0.55	1.06	0.59	3.35	0.46	1.52	1.97
	Piney Creek	4.12	2.64	4.83	1.23	1.46	3.03	1.19	1.80	0.55	1.00	0.56	3.11	0.47	1.47	1.74
	Casselman River	3.75	2.24	4.42	1.09	1.64	2.86	1.24	1.78	0.51	1.07	0.55	3.06	0.51	1.57	1.69
	Emory River	3.82	1.92	4.25	0.90*	2.05*	2.75	1.35	1.85	0.48	1.17	0.57	3.17	0.54	1.71	1.79
	Youghiogheny River	4.57	2.24	5.33	0.96*	1.88	2.78	1.31	1.82	0.51	1.07	0.55	3.09	0.51	1.58	1.71
	Daddy's Creek	4.13	2.18	4.71	1.01	1.82	2.84	1.29	1.83	0.51	1.10	0.56	3.14	0.51	1.61	1.76
	Little Mahoning Creek	4.07	2.80	4.85	1.30	1.36	3.08	1.15	1.78	0.57	0.97	0.55	3.08	0.46	1.43	1.71
	Allegheny River	4.47	2.37	5.23	1.04	1.74	2.85	1.27	1.81	0.53	1.05	0.56	3.09	0.50	1.54	1.72
Marani et al. (1991)	Hacking River	4.81	2.97	5.35	1.30	1.44	3.17	1.18	1.87	0.58	0.99	0.58	3.27	0.44	1.43	1.89
	Beech Creek	3.69	2.61	4.05	1.37	1.36	3.24	1.15	1.87	0.55	1.05	0.58	3.27	0.46	1.51	1.89
	Vermillon River	3.11	2.07	2.80	1.41	1.56	3.62	1.22	2.20*	0.50	1.27	0.64	3.76	0.47	1.76	2.39
	Kaskaska River	3.76	2.63	4.35	1.32	1.37	3.12	1.16	1.80	0.55	1.01	0.56	3.14	0.47	1.48	1.77
	Sagamon River	3.13	1.82	3.29	1.01	1.91	2.92	1.31	1.92	0.47	1.23	0.58	3.30	0.54	1.77	1.92
	Daddy's Creek	4.10	2.18	4.71	1.01	1.81	2.83	1.29	1.82	0.51	1.09	0.56	3.12	0.51	1.60	1.74
	Davison River	3.96	2.41	4.80	1.12	1.56	2.88	1.22	1.75	0.53	1.02	0.55	3.01	0.50	1.52	1.64
	Querecual	4.20	1.75	4.50	0.74*	2.56*	2.65	1.44	1.91	0.46	1.23	0.57	3.24	0.55	1.79	1.86
	Ilice Creek	2.70	2.00	5.10	0.85*	1.43	2.07	1.18	1.22	0.49	0.86	0.42	2.10	0.68	1.43	0.89
	Virginio Creek	3.90	2.30	4.50	1.11	1.63	2.92	1.24	1.81	0.52	1.07	0.56	3.12	0.50	1.57	1.74
	Bisenzio	4.10	2.30	4.60	1.09	1.69	2.94	1.26	1.85	0.52	1.08	0.57	3.19	0.50	1.58	1.81
	Elsa	4.40	1.80	4.20	0.82*	2.52*	2.88	1.43	2.06*	0.47	1.30	0.61	3.55	0.52	1.84	2.18
	Sieve	4.90	2.50	4.60	1.20	1.73	3.28	1.27	2.08*	0.54	1.16	0.63	3.69	0.44	1.61	2.32
Rosso et al. (1991)	Rio Gallina	3.04	2.03	3.92	1.04	1.57	2.66	1.22	1.63	0.49	1.05	0.52	2.84	0.56	1.60	1.48
	Maroggia	3.51	2.02	4.07	1.00	1.79	2.79	1.28	1.79	0.49	1.12	0.55	3.08	0.54	1.65	1.71
	Petrace	4.10	2.10	4.50	0.99*	1.90	2.86	1.31	1.88	0.50	1.14	0.57	3.22	0.51	1.65	1.84
	Arno	4.70	2.50	5.20	1.11	1.69	2.99	1.26	1.88	0.54	1.06	0.57	3.24	0.47	1.53	1.86
	Big	3.24	2.52	4.60	1.21	1.27	2.75	1.12	1.54	0.54	0.92	0.50	2.67	0.53	1.42	1.34
	Big Piney	4.25	3.01	6.32	1.20	1.31	2.76	1.14	1.57	0.58	0.84	0.49	2.61	0.50	1.30	1.28
	Blackwater	3.31	1.85	4.20	0.86*	1.95	2.53	1.32	1.67	0.47	1.10	0.52	2.85	0.58	1.67	1.50
	Bourbeuse	4.12	3.34	6.47	1.29	1.17	2.81	1.08	1.52	0.61	0.78	0.48	2.52	0.49	1.23	1.21
	Gasconade	4.18	3.11	5.83	1.29	1.26	2.91	1.12	1.62	0.59	0.86	0.51	2.75	0.48	1.31	1.40
	Lamine	2.98	1.90	4.08	0.91*	1.70	2.47	1.26	1.55	0.48	1.04	0.50	2.69	0.60	1.61	1.36
	Meramec	3.19	2.18	4.08	1.11	1.49	2.76	1.20	1.65	0.51	1.03	0.53	2.87	0.54	1.55	1.51
	Moreau	3.46	2.98	5.58	1.27	1.14	2.71	1.06	1.44	0.58	0.80	0.47	2.45	0.52	1.28	1.15

* Anomalous Values of Fractal Dimensions.

### 4.2 Fractal relations to shape of hydrologic responses

In order to investigate the relations of $d_{w}$ and $d_{s}$ to hydrologic responses, we use the dimensionless characteristic parameters expressed in Eq. (37) and Eq. (38) of GIUH including $\beta_{G}$ in Eq. (30)

(37) $${\overset{ˆ}{h}}_{p}=h_{p}\left(\frac{L_{\Omega }}{v}\right)=1.31R_{L}^{0.43}$$

(38) $${\overset{ˆ}{t}}_{p}=t_{p}\left(\frac{v}{L_{\Omega }}\right)=0.44{\left(\frac{R_{B}}{R_{A}}\right)}^{0.55}R_{L}^{-0.38}$$

where ${\overset{ˆ}{h}}_{p}$ and ${\overset{ˆ}{t}}_{p}$ correspond to the dimensionless coordinates of the peak for GIUH, where the former amounts to the product of $h_{p}$ and $t_{\Omega }$ ($=L_{\Omega }/v$), whereas the latter is the ratio of $t_{p}$ to $t_{\Omega }$. Furthermore, we also use the dimensionless geomorphologic parameters expressed in Eq. (39) and Eq. (40) which are associated with Nash model including *n* in Eq. (31)

(39) $$\overset{ˆ}{k}=k\left(\frac{v}{L_{\Omega }}\right)=0.70{\left(\frac{R_{A}}{R_{B}R_{L}}\right)}^{0.48}$$

(40) $${\overset{ˆ}{t}}_{L}=t_{L}\left(\frac{v}{L_{\Omega }}\right)=2.30{\left(\frac{R_{B}}{R_{A}}\right)}^{0.3}R_{L}^{-0.41}$$

where $\overset{ˆ}{k}$ and ${\overset{ˆ}{t}}_{L}$ stand for the dimensionless scale parameter of Nash model and the dimensionless basin lag time respectively, in which the former indicates the ratio of *k* to $t_{\Omega }$, while the latter represents the ratio of $t_{L}$ to $t_{\Omega }$. Table 1 contains all of the characteristics which are estimated for a total of thirty-seven channel networks in this study.

Based on Eq. (19), *D* in Eq. (6) can be rewritten with respect to the dynamic transport properties of channel networks

(41) $$D=d_{w}\frac{d_{s}}{2}$$

Furthermore, following the assumption of Rosso (1984) that $\beta =\beta_{G}$, Eq. (36) can be also extended to

(42) $$n\frac{{\left(n-1\right)}^{n}}{\Gamma \left(n\right)}e^{-\left(n-1\right)}\approx D$$

Eq. (42), along with Eq. (6) and Eq. (41), could serve as an inference to the shape of hydrologic responses from the viewpoint of both static and dynamic fractal structures of channel networks. In other words, through Eq. (42), we could directly estimate the value of *n* according to the static structure of channel networks (Eq. (6)) and, then, interpret its hydrologic implication in terms of the dynamic transport properties of channel networks (Eq. (41)). So, we also try to verify Eq. (42) along with the validation of Liu's conjecture presented in the last section for several channel networks in Korea.

**Figure 1:**
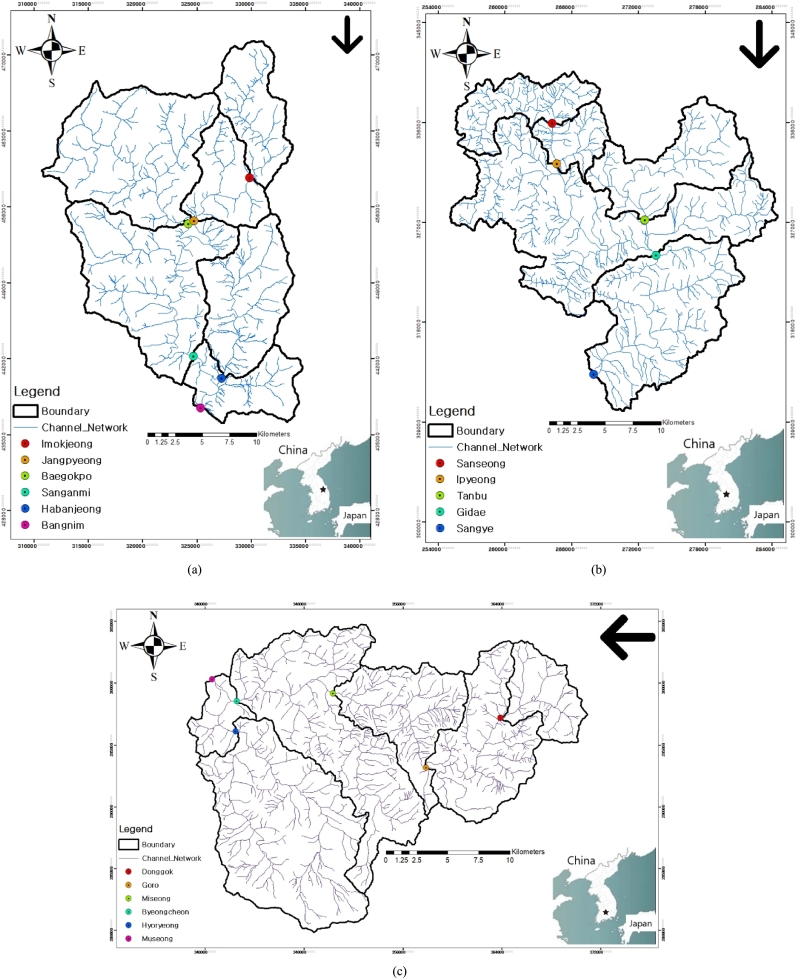
Drainage Maps of the IHP Experimental Basins in Korea: (a) The Pyeongchang River Basin, (b) The Bocheong Creek Basin, (c) The Wi Creek Basin. The arrow in the upper right corner of each figure indicates the downstream direction.

Our case study is concerned with seventeen channel networks which are nested in the three IHP (International Hydrological Programme) experimental basins in Korea as depicted in Fig. 1. A brief overview of the three basins of interest is as follows; The Pyeongchang river basin has a relatively high altitude with a median of 610 m above the mean sea level with the average basin slope being 0.333 by Horton's intersection method, in which the channels tend to be steep upstream but to get mild downstream. The Bocheong creek basin largely consists of forest, of which the altitude in the vicinity of the basin boundary is high whereas the flood plains around the channel networks are flat. A median altitude is 220 m above the mean sea level with about 90% of the drainage area being below 600 m above the mean sea level. The Wi creek basin has a median of 221 m above the mean sea level with the average basin slope being 0.337 by Horton's intersection method.

The channel networks in Fig. 1 are coming from the Blue Line layer of the digital terrain map by Korean National Geographic Information Institute (NGII) at the scale of 1:25,000. Representing channel networks precisely is one of the fundamental tasks for clarifying the geomorphology-based rainfall-runoff mechanisms in catchment hydrology. A number of methods have been developed to extract drainage networks based on DEM so far. These methodologies can be classified into two main types in the application criterion used to distinguish between hillslope and channel components: area threshold (O'Callaghan and Mark, 1984; Tarboton et al., 1992) and slope-area threshold (Montgomery and Foufoula-Georgiou, 1993). However, despite these diverse approaches to the identification of drainage networks with DEM, researchers have not yet reached at a consensus on this issue. Therefore, in this study, we use the Blue lines in Fig. 1 to reduce the uncertainty associated with drainage network delineation. Usually, channel networks such as the ones in Fig. 1 are built on the basis of the field survey. So, we regard those as the extent of the perennial channel networks in this study. Table 2 details those seventeen channel networks in a similar way to Table 1. DEM for the basins of interest is generated in the resolution of 20×20 m by using the digital terrain map of NGII aforementioned. TauDEM (Tarboton, 2003), operated on ArcMap, is utilized to manipulate DEM. For more details on the three IHP experimental basins in Korea, one can refer to http://www.ihpkorea.or.kr/.

**Table 2:** The Channel Networks of the IHP Experimental Basins in Korea.

Basin	Subbaisn	*A* (km^2^)	*R*_*B*_	*R*_*L*_	*R*_*A*_	*d*_*L*_	*d*_*N*_	*d*_*w*_	*d*_*s*_	*D*	${\overset{ˆ}{h}}_{p}$	${\overset{ˆ}{t}}_{p}$	*β*_*G*_	*n*	$\overset{ˆ}{k}$	${\overset{ˆ}{t}}_{L}$	*nβ*_*G*_
Pyungchang	Imokjeong	55.80	3.58	2.43	4.18	1.24	1.44	3.02	1.18	1.78	0.53	1.04	0.56	3.10	0.49	1.53	1.73
	Jangpyeong	105.14	4.53	2.96	5.20	1.32	1.39	3.15	1.16	1.83	0.58	0.97	0.57	3.19	0.44	1.42	1.81
	Baegokpo	143.84	3.36	1.83	3.85	0.90*	2.01*	2.69	1.33	1.80	0.47	1.17	0.55	3.09	0.56	1.73	1.71
	Sanganmi	393.73	4.12	2.53	4.73	1.19	1.53	3.02	1.21	1.82	0.54	1.03	0.56	3.15	0.48	1.51	1.78
	Habanjeong	85.56	4.25	2.29	4.93	1.04	1.75	2.85	1.27	1.81	0.52	1.07	0.56	3.11	0.51	1.57	1.73
	Bangnim	527.90	4.46	2.66	5.07	1.21	1.53	3.05	1.21	1.84	0.55	1.02	0.57	3.19	0.47	1.48	1.81
Bocheong	Sanseong	49.10	3.88	2.42	4.59	1.16	1.53	2.94	1.21	1.78	0.53	1.03	0.55	3.07	0.50	1.52	1.70
	Ipyeong	76.30	4.49	2.67	5.27	1.18	1.53	2.99	1.21	1.81	0.56	1.00	0.56	3.11	0.47	1.47	1.73
	Tanbu	77.51	4.44	2.91	5.35	1.27	1.40	3.05	1.17	1.78	0.58	0.95	0.55	3.07	0.46	1.41	1.69
	Gidae	345.14	3.42	1.23	3.86	0.31*	5.94*	2.13	1.71	1.82	0.40	1.37	0.55	3.04	0.67	2.04	1.67
	Sangye	485.21	3.68	1.98	4.03	0.98*	1.91	2.85	1.31	1.87	0.49	1.16	0.57	3.22	0.53	1.69	1.84
Wi	Donggok	33.39	3.69	2.25	4.52	1.08	1.61	2.81	1.23	1.73	0.52	1.04	0.54	2.97	0.52	1.55	1.61
	Goro	109.04	3.59	1.99	4.07	0.98*	1.86	2.80	1.30	1.82	0.49	1.14	0.56	3.13	0.53	1.67	1.75
	Miseong	171.64	4.04	2.32	4.37	1.14	1.66	3.03	1.25	1.89	0.52	1.10	0.58	3.28	0.49	1.59	1.90
	Byeongcheon	302.95	4.60	2.60	5.03	1.18	1.60	3.07	1.23	1.89	0.55	1.05	0.58	3.28	0.46	1.52	1.90
	Hyoryeong	150.38	4.13	1.63	4.72	0.63*	2.90*	2.46	1.49	1.83	0.45	1.22	0.55	3.07	0.59	1.81	1.69
	Museong	472.58	3.94	1.62	4.42	0.65*	2.84*	2.49	1.48	1.85	0.45	1.24	0.56	3.11	0.59	1.83	1.74

* Anomalous Values of Fractal Dimensions.

## 5 Results and discussion

### 5.1 Validation of Liu's conjecture

**Figure 2:**
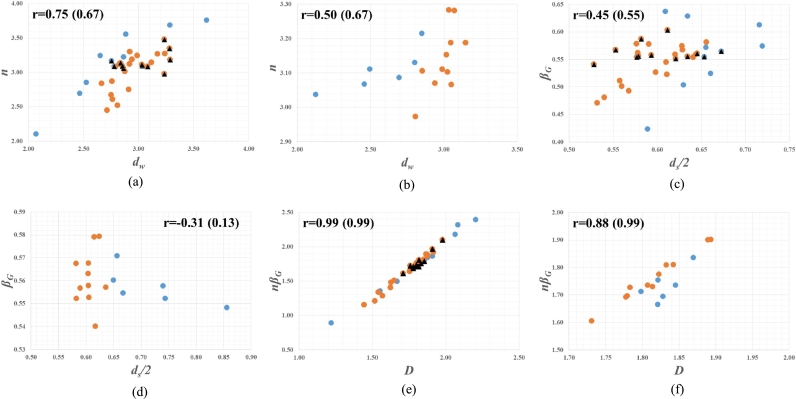
Validation of Liu's Conjecture: (a), (c), and (e) illustrate the results from the data set in Table 1, where the triangles in black belong to Morisawa (1962) data while the remaining falls to the data of Marani et al. (1991) as well as Rosso et al. (1991) among which the dots in blue represent the anomalous values marked by * in Table 1; (b), (d), and (f) demonstrate the results from the data set in Table 2, where the dots in blue correspond to the anomalous values marked by * in Table 2; The correlation coefficient r is inserted into each figure, in which the number in parenthesis denotes the value of r for the data excluding dots in blue.

Fig. 2 demonstrates the validation of Liu's conjecture in Eq. (34) to Eq. (36) on the basis of the two separate data sets in Table 1 and Table 2. In this figure, Fig. 2 (a), Fig. 2 (c), and Fig. 2 (e) represent the results from the data set in Table 1, where the triangles in black belong to Morisawa (1962) data while the remaining falls to the data of Marani et al. (1991) as well as Rosso et al. (1991) among which the dots in blue denote the anomalous values marked by * in Table 1. Fig. 2 (b), Fig. 2 (d), and Fig. 2 (f) illustrate the results from the data set in Table 2, where the dots in blue correspond to the anomalous values marked by * in Table 2. Furthermore, the correlation coefficient r is inserted into each figure, in which the number in parenthesis denotes the value of r for the data excluding the dots in blue.

If Liu's conjecture is valid, all of the plotting points in each figure of Fig. 2 should be placed on a correspondence line with an angle of 45°. However, we couldn't find that kind of behavior in this figure. Especially, in the case of Fig. 2 (a) to Fig. 2 (d), there couldn't be found any specific trend between *n* and $d_{w}$ as well as $\beta_{G}$ and $d_{s}/2$ even with a limited inspection of only triangles in black. In this regard, one can raise questions about Liu's conjecture in Eq. (34) and Eq. (35). In the hydrologic sense, both of *n* and $\beta_{G}$ are expected to have a close relation to the shape of hydrologic responses, following the assumption of Rosso (1984) that $\beta =\beta_{G}$. However, $d_{w}$ and $d_{s}$ represent separate dynamic transport properties of channel networks as evident in Eq. (20) and Eq. (21). So, it would be irrational that two parameters, *n* and $\beta_{G}$ which show a qualitatively same attribute, have different sources, $d_{w}$ and $d_{s}$ individually, as in Eq. (34) and Eq. (35). In fact, without any inspection of plotting such as Fig. 2, Liu (1992) just compares the average values of *n* and $\beta_{G}$ with the ones of $d_{w}$ and $d_{s}/2$ respectively, to find the two pairs of those averages being of similar magnitude. Accordingly, it seems not reasonable that the first two in Liu's conjecture (Eq. (34) and Eq. (35)) are considered as a general relation to the shape of hydrologic responses.

However, it can be observed from Fig. 2 (e) and Fig. 2 (f) that $n\beta_{G}$ is closely related to *D* as follows

(43) $$n\beta_{G}\propto D$$

Eq. (43) implicitly states that only Eq. (36), the third conjecture of Liu for $n\beta_{G}$, might be valid. Following the rationale aforementioned, it could be also inferred that the relation in Eq. (43) doesn't stem from the product of Eq. (34) and Eq. (35). Rather, $n\beta_{G}$ might be considered to reflect the composite nature of hydrologic responses in terms of *D* as the product of $d_{w}$ and $d_{s}/2$. So, we could say that Liu's conjecture stresses the combined effects of dynamic transport properties of channel networks on the shape of hydrologic responses. It is also noted that the three dots in blue, which are deviated from the clear trend in Fig. 2 (f), are directly related to the anomalous values of fractal dimensions with * in Table 2. Accordingly, we could be sure that Eq. (43) tends to be valid at least within the plausible range of fractal dimensions for channel networks.

### 5.2 Fractal relations to peak of GIUH

**Figure 3:**
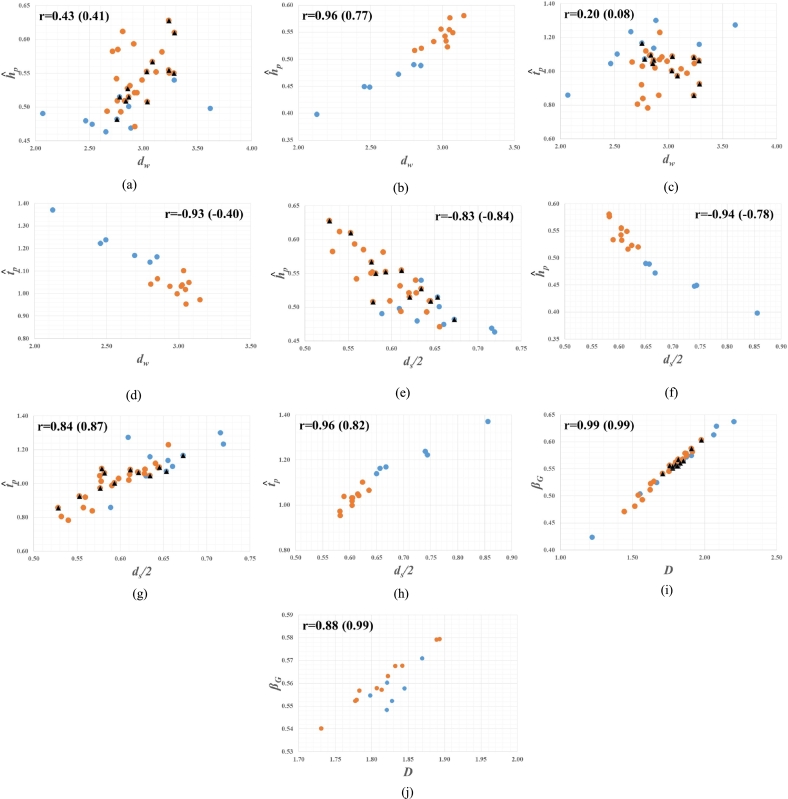
Fractal Relations to the Dimensionless Peak of GIUH: (a), (c), (e), (g), and (i) illustrate the results from the data set in Table 1, where the triangles in black belong to Morisawa (1962) data while the remaining falls to the data of Marani et al. (1991) as well as Rosso et al. (1991) among which the dots in blue represent the anomalous values marked by * in Table 1; (b), (d), (f), (h), and (j) demonstrate the results from the data set in Table 2, where the dots in blue correspond to the anomalous values marked by * in Table 2; The correlation coefficient r is inserted into each figure, in which the number in parenthesis denotes the value of r for the data excluding dots in blue.

Fig. 3 shows the significant relations between the dimensionless characteristic parameters of GIUH (${\overset{ˆ}{h}}_{p}$, ${\overset{ˆ}{t}}_{p}$, and $\beta_{G}$) and the dynamic transport properties of channel networks ($d_{w}$ and $d_{s}/2$ including *D*), based on the two separate data sets in Table 1 and Table 2. This figure is depicted in the same manner as Fig. 2 (see the caption of Fig. 3 for more in details).

There can be seen that the dimensionless peak of GIUH (hydrologic responses in the broader sense) has a relatively close relation to $d_{s}/2$ rather than $d_{w}$. Especially, it can be consistently observed from Fig. 3 (e) to Fig. 3 (h) that ${\overset{ˆ}{t}}_{p}$ is proportional to $d_{s}/2$ while ${\overset{ˆ}{h}}_{p}$ vice versa as follows

(44) $${\overset{ˆ}{h}}_{p}\propto {\left(\frac{d_{s}}{2}\right)}^{-1}$$

(45) $${\overset{ˆ}{t}}_{p}\propto \frac{d_{s}}{2}$$

Despite the absence of scale parameters such as *k*, *v*, and $L_{\Omega }$, Eq. (44) and Eq. (45) are reminiscent of Eq. (23) and Eq. (24) as well as Eq. (28) and Eq. (29). Nevertheless, it is also noticed from Fig. 3 (b) and Fig. 3 (d) that the dimensionless peak of GIUH has high correlations with $d_{w}$, for the channel networks in Korea (i.e. the data set in Table 2). However, when excluding the dots in blue in Fig. 3 (d), the value of r reduces significantly lower indicating that $d_{w}$ has a lesser effect on hydrologic responses than $d_{s}$. So, we could expect that the connectivity of channel networks has important hydrological implications as earlier recognized by Liu (1992) in that the peak of IUH has long been thought one of the most important characteristics of hydrologic responses (Henderson, 1963). Moreover, it is well known that $d_{s}/2$ is intimately related to the concept of compact visitation (Sokolov, 2012) which refers to a property of random walks to visit practically all the sites within the domain of $L_{\|}$. Therefore, it makes sense that ${\overset{ˆ}{t}}_{p}$ along with ${\overset{ˆ}{h}}_{p}$ has a close relation to $d_{s}/2$ in the context of random walks.

Interestingly, it can be observed from Fig. 3 (i) and Fig. 3 (j) that $\beta_{G}$ has a close relation with *D* similar to $n\beta_{G}$ in Eq. (43)

(46) $$\beta_{G}\propto D$$

As mentioned before, $\beta_{G}$ as well as *β* can be regarded as a salient characteristic for the shape of hydrologic responses. We can also find that the three points with the anomalous values of fractal dimensions deviate from the clear trend in Fig. 3 (j), the same as in Fig. 2 (f). This implies that $\beta_{G}$ has the same meaning as $n\beta_{G}$ as expected before. So, based on Eq. (46), we could consider that the fractal dimension of channel networks, *D*, has a kind of combined effects on shaping hydrologic responses at the basin scale, reflecting the conductivity and connectivity of channel networks.

### 5.3 Fractal relations to Nash model

**Figure 4:**
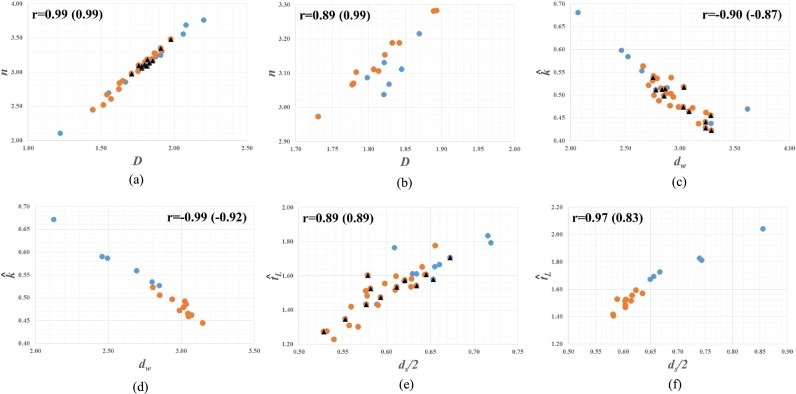
Fractal Relations to Nash Model: (a), (c), and (e) illustrate the results from the data set in Table 1, where the triangles in black belong to Morisawa (1962) data while the remaining falls to the data of Marani et al. (1991) as well as Rosso et al. (1991) among which the dots in blue represent the anomalous values marked by * in Table 1; (b), (d), and (f) demonstrate the results from the data set in Table 2, where the dots in blue correspond to the anomalous values marked by * in Table 2; The correlation coefficient r is inserted into each figure, in which the number in parenthesis denotes the value of r for the data excluding dots in blue.

Fig. 4 shows the three significant relations between the dimensionless geomorphologic parameters of Nash model (*n*, $\overset{ˆ}{k}$, ${\overset{ˆ}{t}}_{L}$) and the dynamic transport properties of channel networks ($d_{w}$ and $d_{s}/2$ including *D*), based on the two separate data sets in Table 1 and Table 2. This figure is depicted in the same manner as Fig. 2 and Fig. 3 (see the caption of Fig. 4 for more in details) and can be read as follows

(47) n∝D

(48) $$\overset{ˆ}{k}\propto d_{w}^{-1}=\frac{1}{D}\left(\frac{d_{s}}{2}\right)$$

(49) $${\overset{ˆ}{t}}_{L}\propto \frac{d_{s}}{2}$$

At first, from Eq. (49), ${\overset{ˆ}{t}}_{L}$ is noticed to have a close relation with $d_{s}/2$, similarly to ${\overset{ˆ}{t}}_{p}$ as presented in the last section. Both the time to peak of IUH and the basin lag time have been well known as the most critical characteristic times of the hydrologic responses to rainfall (Singh, 1988). Accordingly, we could be sure that the connectivity of channel networks would have an intimate relationship with the characteristic times of hydrologic responses. It can be also seen in Eq. (47) that *n* is closely related to *D* similar to $n\beta_{G}$ in Eq. (43) and $\beta_{G}$ in Eq. (46). Furthermore, the same as in Fig. 2 (f) and Fig. 3 (j), the three points with the anomalous values of fractal dimensions show the deviation from the clear trend in Fig. 4 (b) again. Thereby, *n*, $\beta_{G}$, and $n\beta_{G}$ can be considered as qualitatively the same properties for the shape of hydrologic responses, so Liu's conjecture for $n\beta_{G}$ (Eq. (36)) might be only valid to stress the combined effects of the conductivity and connectivity of channel networks on shaping hydrologic responses at the basin scale. Especially, it is interesting that an interrelation of Eq. (47), Eq. (48), and Eq. (49) is consistent with Eq. (33).

At this stage, it should be considered carefully that $\overset{ˆ}{k}$ in Eq. (48) shows a close relation to $d_{w}$. In fact, from Fig. 2 and Fig. 3, there can be rarely found out any significant relation between $d_{w}$ and the dimensionless characteristics of hydrologic responses used in this study, except for $\overset{ˆ}{k}$. Based on Eq. (11), $d_{w}$ indicates a close connection of the conductivity through porous media to diffusion process (Hendrick and Renard, 2016). So, we could think that, unlike electrical currents or karst networks (Hendrick and Renard, 2016), diffusion process alone might not be sufficient to explain the movement of water particles through channel networks in the context of random walks. Moreover, based on Eq. (20) and Eq. (21), $d_{w}$ and $d_{s}$ indicate separate properties of channel networks although they are interrelated with each other through Eq. (19). Therefore, we suggest the second equality rather than the first proportionality in Eq. (48), for the sake of interpreting $\overset{ˆ}{k}$ in this study as follows: Basically, this parameter can be also regarded as one of the characteristic times of hydrologic responses, which is conditioned by *n* within the framework of Nash model (Eq. (33)). So, it could be reasonable to assume $\overset{ˆ}{k}$ to have a close relation with $d_{s}/2$ similarly to ${\overset{ˆ}{t}}_{p}$ and ${\overset{ˆ}{t}}_{L}$ and, thereby, the second equality in Eq. (48) could be considered as an explanation of a single linear reservoir constrained by *D* in the context of Nash model.

### 5.4 Further discussion on the shape of hydrologic responses

Table 3 lists the regressions for both of $n\beta_{G}$ and *n* to *D* following Eq. (43) and Eq. (47) by using the separate data sets in Table 1 and Table 2, where $R^{2}$ denotes the coefficient of determination. By referencing Eq. (36), the third conjecture of Liu, we choose a linear regression model and, then, perform regression for two types of data depending on the presence of the anomalous values of fractal dimensions with * in Table 1 and Table 2 (denoted by w/* and w/o*; see the caption of Table 3 for more in details). Interestingly, a pair of regressions in the same row have similar regression coefficients each other in Table 3. As already seen before, the data sets in Table 1 and Table 2 have extremely different sources, so Table 3 might be considered to demonstrate the validity of those regressions even though they are in the simplest form of linear regression. The two types of regressions for $n\beta_{G}$ and *n* in Table 3 could be considered equivalent qualitatively, so that they can be used to estimate *n* interchangeably. The only difference is in the need for numerical approach to *n* posed by Eq. (42). Even though a vast of research has been devoted to fractal properties of channel networks so far, the regressions in Table 3 would be the first suggestion for the direct application of the fractal dimension to rainfall-runoff model (more specifically Nash model). Furthermore, based on the results presented in the previous sections, we could infer that the shape of hydrologic responses could be influenced by the static fractal structure of channel networks (Eq. (6)), while it could be also interpreted as a combined result of the conductivity and the connectivity of channel networks (Eq. (41)). So, the suggestion of this study could provide an avenue to direct connection of the shape of hydrologic responses to the form of channel networks.

**Table 3:** The Regressions of *nβ*_*G*_ and *n* to *D*.

	The Published Data Set for the Various Channel Networks		The Channel Networks of the IHP Experimental Basins in Korea	
	Regression	*R*^2^	Regression	*R*^2^
w/*	*nβ*_*G*_ = 1.6459*D* − 1.2216	0.9808	*nβ*_*G*_ = 1.6939*D* − 1.3326	0.7823
w/o*	*nβ*_*G*_ = 1.7460*D* − 1.3985	0.9804	*nβ*_*G*_ = 1.8172*D* − 1.5374	0.9768
w/*	*n* = 1.7893*D* − 0.1152	0.9850	*n* = 1.7692*D* − 0.0941	0.7850
w/o*	*n* = 1.9116*D* − 0.3256	0.9810	*n* = 1.8962*D* − 0.3050	0.9768

w/*: Data Set including the anomalous values of fractal dimensions with * in Table 2 and Table 3.

w/o*: Data Set excluding the anomalous values of fractal dimensions with * in Table 2 and Table 3.

Based on Table 3, it could be also expected that the runoff hydrographs at the outlet of a river basin would be shaped by the fractal dimensions of channel networks in the sense of both the static structures and dynamic transport properties of those, because this kind of signals can be constructed by the proportionality and the superposition of hydrologic responses to rainfall following the linear system theory (Dooge, 1973). However, it is also well known that the time scale of hydrologic responses, related to the flow velocity on hillslopes and within channel networks, is also crucial to analyze the rainfall-runoff process of river basins correctly (Agnese et al., 1988). As mentioned before, in this study, we focus on the validation of Liu's conjecture so that the results are largely concerned with the geomorphology of river basins around channel networks. Therefore, in order to establish more general relations for the shape of hydrologic responses, further research on this topic is required based on the observation of rainfall-runoff processes. Moreover, the additional analysis on the effect of DEM resolutions should be included in the future research because the basin characteristics are well known to be influenced by those. The concept of the equivalent Horton ratios suggested by Moussa (2009) and Kim (2022), which is known to be independent on the observation scale of channel networks, might be valuable tools for the site-specific research.

## 6 Conclusions

The followings are the noteworthy results from this study

1) Through Horton's law of drainage composition, the fractal dimension of channel networks can directly connect the static structure of channel networks to the dynamic transport properties of channel networks within the framework of random walks on fractal networks.
2) The peak of hydrologic responses has a closer relation to the connectivity of channel networks rather than the conductivity of those in the context of the dynamic transport properties of fractal networks. Moreover, the characteristic times of hydrologic responses also tend to relate to the connectivity of channel networks intimately.
3) On one hand, the shape of hydrologic responses would be affected by the branching property of channel networks and sinuosity of individual channel segments in terms of their static structure. On the other hand, the shape of hydrologic responses could be also interpreted as a combined result of the conductivity and the connectivity of channel networks in terms of their dynamic transport property.
4) Thereby, it can be inferred that the runoff hydrographs of a river basin would be shaped by the fractal dimension of its channel network because this kind of signal can be constructed by the proportionality and the superposition of hydrologic responses to rainfall following the linear system theory

## Declarations

### Author contribution statement

Joo-Cheol Kim: Conceived and designed the experiments; Performed the experiments; Analyzed and interpreted the data; Contributed reagents, materials, analysis tools or data; Wrote the paper.

Yeo-Jin Yoon: Analyzed and interpreted the data; Contributed reagents, materials, analysis tools or data; Wrote the paper.

### Funding statement

Dr. Joo-Cheol Kim was supported by 10.13039/501100003725National Research Foundation of Korea [NRF-2022R1I1A1A01056269].

### Data availability statement

Data included in article/supp. material/referenced in article.

### Declaration of interest's statement

The authors declare no conflict of interest.

### Additional information

No additional information is available for this paper.
